# A Neural Network-Accelerated Approach for Orthopedic Implant Design and Evaluation Through Strain Shielding Analysis

**DOI:** 10.3390/biomimetics10040238

**Published:** 2025-04-13

**Authors:** Ana Isabel Lopes Pais, Jorge Lino Alves, Jorge Belinha

**Affiliations:** 1 Department of Mechanical Engineering, Faculty of Engineering, University of Porto, Rua Dr. Roberto Frias, s/n, 4200-465 Porto, Portugal; anapais@fe.up.pt (A.I.L.P.); falves@fe.up.pt (J.L.A.); 2 INEGI—Institute of Science and Innovation in Mechanical and Industrial Engineering, Rua Dr. Roberto Frias, 400, 4200-465 Porto, Portugal; 3 ISEP, Polytechnic of Porto, Rua Dr. António Bernardino de Almeida, n. 431, 4249-015 Porto, Portugal

**Keywords:** orthopedic implants, neural networks, stress shielding, porous implant design

## Abstract

The design of orthopedic implants is a complex challenge, requiring the careful balance of mechanical performance and biological integration to ensure long-term success. This study focuses on the development of a porous femoral stem implant aimed at reducing stiffness and mitigating stress shielding effects. To accelerate the design process, neural networks were trained to predict the optimal density distribution of the implant, enabling rapid optimization. Two initial design spaces were evaluated, revealing the necessity of incorporating the femur’s anatomical features into the design process. The trained models achieved a median error near 0 for both conventional and extended design spaces, producing optimized designs in a fraction of the computational time typically required. Finite element analysis (FEA) was employed to assess the mechanical performance of the neural network-generated implants. The results demonstrated that the neural network predictions effectively reduced stress shielding compared to a solid model in 50% of the test cases. While the graded porosity implant design did not show significant differences in stress shielding prevention compared to a uniform porosity design, it was found to be significantly stronger, highlighting its potential for enhanced durability. This work underscores the efficacy of neural network-accelerated design in improving implant development efficiency and performance.

## 1. Introduction

Orthopedic implants play a crucial role in the treatment of bone fractures, joint replacements, and other orthopedic conditions. The design of these implants is essential to ensure both optimal mechanical performance and biocompatibility. To prevent stress shielding (a phenomenon where bone density decreases due to inclusion of an implant, causing the the abrupt modification of the stress field on the bone tissue), the mechanical properties of the implant must closely match those of the surrounding bone. Stress shielding can adversely affect bone growth and remodeling, which are driven by mechanical stimuli [1], potentially leading to bone resorption and implant loosening.

One approach to address the mismatch in mechanical properties involves carefully selecting implant materials. Titanium alloys, for example, are advantageous due to their Young’s modulus being closer to that of bone compared to materials like stainless steel or cobalt–chromium alloys. Research has explored this issue extensively; for instance, Soliman et al. [2] evaluated an implant design called “Summit”, which incorporated various alloys, including Inconel 718, 316 Stainless Steel, Ti-6Al-7Nb, Ti-6Al-4V, Ti-12Mo-6Zr-2Fe, and Ti-33.6Nb-4Sn. Additional studies have examined materials such as carbon fiber composites, Ti-6Al-4V, 630 stainless steel [3], cobalt–chromium alloys, hydroxyapatite, Ti-29Nb-Ta-Zr, Ti-6Al-4V, and zirconia–hydroxyapatite composites [4] to mitigate stress shielding effects.

Controlled stiffness reduction in implants can be achieved through the use of porous materials. By manipulating the volume fraction and geometry of unit cells, implants can be tailored to achieve specific mechanical and biomechanical properties. Examples include truss-like unit cells [5], unit cells based on triply periodic minimal surfaces (TPMS) with skeletal-like geometries [6], and TPMS with sheet-like geometries [7]. Scaffolds exemplify this strategy by reducing mechanical stiffness and increasing surface area to encourage bone growth [8].

The development of femoral stem implants can be enhanced by achieving the required mechanical properties through the inclusion of porous structures [9]. Advances in additive manufacturing (AM) processes have made it possible to construct these intricate structures with high precision. For example, the studies of Mehboob et al. [10], Cortis et al. [11], Cheah et al. [12], Abate et al. [13] explored different truss-like unit cells to create porous femoral stem designs, namely the body-centered cubic (BCC) [10,11], the FBCCz and octet [12], and ventile lattices [13]. Other studies focused on the use of TPMS structures such as the gyroid and the primitive used in the works of Moghariya and Gurrala [14] and Salaha et al. [15]. In the latter, the TPMS structure was compared to a Voronoï porous structure. Although the conclusions of all these works are dependent on the unit cell porosity, all these studies consensually show that controlling the mechanical properties of the femoral stem via the addition of porous structures is beneficial against the prevention of stress shielding and micromotion of the implant.

All of the mentioned works consider the uniform porosity of the structure. Nevertheless, it is possible to enhance the behavior of the stem by spacial variation or the gradation of its properties, for example, through variation in the density of unit cells. Some researchers developed graded porosity implant designs through a linear or radial gradient of porosity. Rahmat et al. [16] axially increased and decreased the density of several TPMS, namely gyroid and IWP sheet- and truss-like unit cells, and found that a decreasing gradient is better to reduce stress shielding. On the other hand, Zoubi et al. [17] varied the porosity of cubic unit cells radially. They found that a 70% average porosity led to the most similar properties in comparison to intact bone. In a different work, Wang et al. [18] increased and decreased the density of diamond unit cells axially and radially.

In 2008, Harrysson et al. [19] identified the potential of optimization algorithms in the development of non-stochastic mesh femoral stem prosthesis. Some works combine structural optimization techniques to predict the density distributions of the stem. The approaches sometimes include homogenization techniques to create scaling laws that relate the unit cell porosity to the macroscale properties. One example of this is the work of Müller et al. [20], in which the optimization of a femoral stem was based on the BCC cell. In their approach, the stem porosity was minimized to reduce its energy stiffness, subject to a stress constraint to ensure that the stress verified in the bone after implantation is closer to the intact femur, and this effectively reduced the stress shielding effect. Ziaie et al. [21] used a Solid Isotropic Material with Penalization (SIMP) approach where the density distribution was then rendered considering two TPMS sheet-like unit cells, the gyroid and the diamond. The results showed that gyroid structures are more effective in the reduction of stress shielding. Finally, while uniformly porous structures were more effective in stress shielding reduction, only graded porosity structures would fulfill mechanical strength requirements. Tan and van Arkel [22] compared a stochastic porous implant model to a hollowed implant obtained with the method of SIMP and concluded that while both presented very similar mechanical properties, the stochastic porosity can result in higher tolerance to unanticipated loading conditions. The work of Kladovasilakis et al. [23] created a functional gradation of TPMS strcutures based on a FEM analysis of the solid implant to increase or decrease density according to the most stressed areas.

Neural networks (NNs) are increasingly being recognized as robust tools for accelerating the design of implants while simultaneously achieving higher performance or greater levels of customization. In the research conducted by Cilla et al. [24], machine learning (ML) methodologies, specifically support vector machines (SVM) and NNs, are utilized to optimize implant design with the aim of minimizing stress shielding. In this methodology, geometrical parameters act as the design variables, and the objective variable is the difference in principal strains measured before and after the insertion of the implant. In a similar manner, Chanda et al. [25] enhanced implant design through the use of artificial neural networks (ANNs), focusing on primary stability, which is crucial prior to the commencement of osteointegration. This stability is mainly affected by the micromotion between the bone and the implant. The study employed 18 input variables related to the geometry of the femoral stem design, with the output variable representing an instability index. Subsequently, a genetic algorithm (GA) was used to optimize the design based on the ANN model.

Further expanding on the optimization of implant design, Ghosh et al. [26] investigated the relationship between bone growth and the microtexture of implants. An NN was used to model bone growth levels as a function of geometrical parameters across three different models. A GA was then applied to develop optimized microtextures that promote enhanced bone growth. Utilizing a similar approach, Roy et al. [27] employed ANNs to predict stress and strain in dental implants based on bone and implant geometry parameters, effectively eliminating the need for the finite element method (FEM) analysis phase. A GA was subsequently applied to identify the optimal geometry for the implant.

To summarize, ANNs are highly effective for modeling implant properties as functions of parameters such as geometry or bone/tissue characteristics. By integrating these models with classical optimization techniques, optimal implant designs can be achieved.

Examining the previously mentioned strategies, there remains a notable gap in the literature regarding the application of NNs as complete surrogate frameworks for the implant design process. In this study, NNs are employed to predict the optimal foam density distribution required for the design of a porous femoral stem implant. The challenge of identifying the optimal density distribution is analogous to a structural optimization problem, where the objective is to devise a new structural design that meets specific criteria.

Several instances in the literature have employed machine learning (ML) and deep learning (DL) methodologies to identify an optimal structural configuration. For example, Sosnovik and Oseledets [28] utilized convolutional neural networks (CNNs) in conjunction with image segmentation techniques to forecast the final optimal configuration using the solid isotropic material with penalization (SIMP) method, beginning from an intermediate phase of the optimization process. Another example of applying DL approaches for partial solutions is the study by Oh et al. [29], who used generative adversarial networks (GANs) to create an initial estimate, which was then refined through traditional topology optimization (TO) algorithms.

To decrease computational costs, some researchers have developed direct mappings between initial conditions and the ultimate structural configuration. For example, Zheng et al. [30] mapped the final structure based on applied loads and boundary conditions, while Yan et al. [31] established a direct mapping between the principal stress field at the initial iteration and the final density. Taking an alternative approach, Chandrasekhar and Suresh [32] exploited the properties of feed-forward neural networks (FNNs) to model the problem by incorporating the SIMP interpolation scheme as the neural network’s activation functions. Consequently, the optimization process involves minimizing the network’s loss function, similar to conventional methods where structural compliance is minimized.

This study highlights the importance of achieving an optimal density distribution in femoral stem implants while addressing the high computational cost associated with structural optimization due to its iterative nature. To mitigate these challenges, an accelerated approach is proposed, leveraging neural networks to streamline the design process. The neural network is trained on a dataset of optimized gyroid foam implants, where the density distribution serves as the output variable and implant geometrical features serve as the input variables. Once trained, the neural network predicts the optimal density distribution for new implant designs. These predictions are subsequently evaluated through finite element analysis to assess the implant’s mechanical performance, with a particular emphasis on minimizing stress shielding effects.

## 2. Materials and Methods

### 2.1. Bio-Inspired Remodeling Algorithm

The dataset used in the training of the neural networks consisted of several optimized gyroid foam implants in order to achieve optimal density distribution. The optimized design is obtained with a bio-inspired remodeling algorithm integrated with a finite element solver for the linear elasto-static problem at each iteration. The algorithm is based on the bone remodeling process, where the bone adapts to the mechanical environment by decreasing or growing in accordance to the mechanical stimulus. The algorithm is based on the following steps:

1. The initial implant design is created. This represents the design domain as well as any part of the domain that must remain solid (and therefore not subject to remodeling).
2. The mechanical properties of the foam material are defined based on its apparent density from the density law as follows:
  (1) $$E\left[MPa\right]=f_{1}\left(v_{f}\right)\cdot E_{s}\left[MPa\right],$$
  which were defined by the homogenization of the foam material [33], where $E_{s}$ is the Young’s modulus of the solid material, *E*, and $f_{1}$ is a function dependent on the bone tissue volume fraction, $v_{f}$,
  (2) $$f_{1}:\frac{E^{*}}{E_{s}}=0.1018{\left(v_{f}\right)}^{3}+0.4388{\left(v_{f}\right)}^{2}+0.2405\left(v_{f}\right).$$
  The superscript ∗ denotes the homogenized property.
3. Loads and boundary conditions are applied to the model. For this case, the implant was fixed at the distal-most part and a force was applied at the taper.
4. The stress distribution in the implant is calculated using finite element analysis:
  (a) The stiffness matrix K is calculated, and the displacement field obtained:
    $$u=K^{-1}f$$
    where u is the global nodal displacement vector, f is the global force vector, and K is the global stiffness matrix.
  (b) The strain ε is calculated
    ε=Lu
    where L is the differential operator, u is the nodal displacement vector, and ε is the strain tensor in Voigt notation.
  (c) The stress σ is calculated
    σ=cε
    where c is the material constitutive matrix, ε is the strain tensor, and σ is the stress tensor, all in Voigt notation.
5. The points subject to remodeling are chosen according to stress criteria. The $n_{inc}=\alpha *n_{intpoints}$ points where the equivalent von Mises stress $\sigma^{VM}$ is the highest will have their density increased and the $n_{red}=\beta *n_{intpoints}$ points where the equivalent von Mises stress $\sigma^{VM}$ is the lowest will have their density decreased.
  (3) $$\sigma^{VM}=\sqrt{\frac{{\left(\sigma_{xx}-\sigma_{yy}\right)}^{2}+{\left(\sigma_{yy}-\sigma_{zz}\right)}^{2}+{\left(\sigma_{zz}-\sigma_{xx}\right)}^{2}+6\left(\tau_{xy}^{2}+\tau_{yz}^{2}+\tau_{zx}^{2}\right)}{2}}$$
6. The volume fraction of the points subject to remodeling is updated based on the stress distribution from $\rho_{app}=g_{2}\left(\sigma \left[MPa\right]/\sigma_{s}\left[MPa\right]\right)$; the inverse of $g_{1}$ is also obtained by the homogenization of the foam material [33], where $\sigma_{s}$ is the yield stress of the solid material and $\sigma^{*}$ is the apparent yield stress of the foam material.
  (4) $$g_{1}:\frac{\sigma^{*}}{\sigma_{s}}=0.5545{\left(v_{f}\right)}^{3}+0.1250{\left(v_{f}\right)}^{2}+0.4666\left(v_{f}\right)$$
7. The process is repeated from item 2, until either convergence or a control average density is achieved.

The solid material considered for this work was Ti-6Al-4V with a Young’s modulus of 110 GPa and a yield stress of 850 MPa.

This approach will reduce the overall mass of the design domain, and therefore reduce stiffness without significantly reducing the strength of the part. Therefore, this methodology is a valid approach to design porous implants featuring the necessary mechanical requirements for orthopedic applications while taking into account the stress shielding effect.

### 2.2. Implant Models

**Figure 1:**
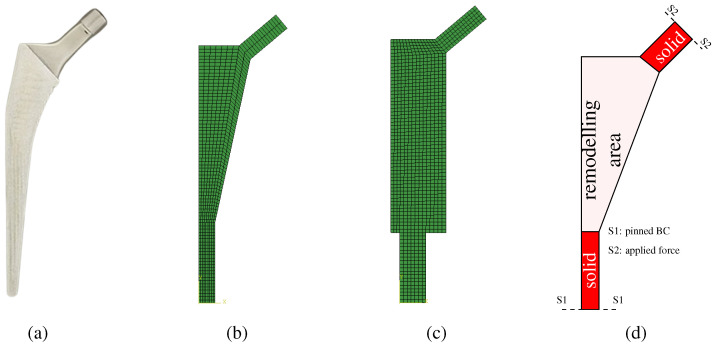
Implant models: (**a**) example of an exiting femoral stem implant, (**b**) conventional model meshed, (**c**) extended model meshed, (**d**) solid parts and foam parts of the design domain showing the boundary condition locations.

Two implant models were considered at this stage. The first model is called the conventional implant design, while the second model will be called the extended design. The conventional model, Figure 1b, follows the overall geometry shown in Figure 1a. The extended model has a larger design space, as it can be seen in Figure 1c. The objective of the extended model is to attest if the optimization procedure can obtain the same solution as the conventional model, which is constrained to the anatomical features.

**Figure 2:**
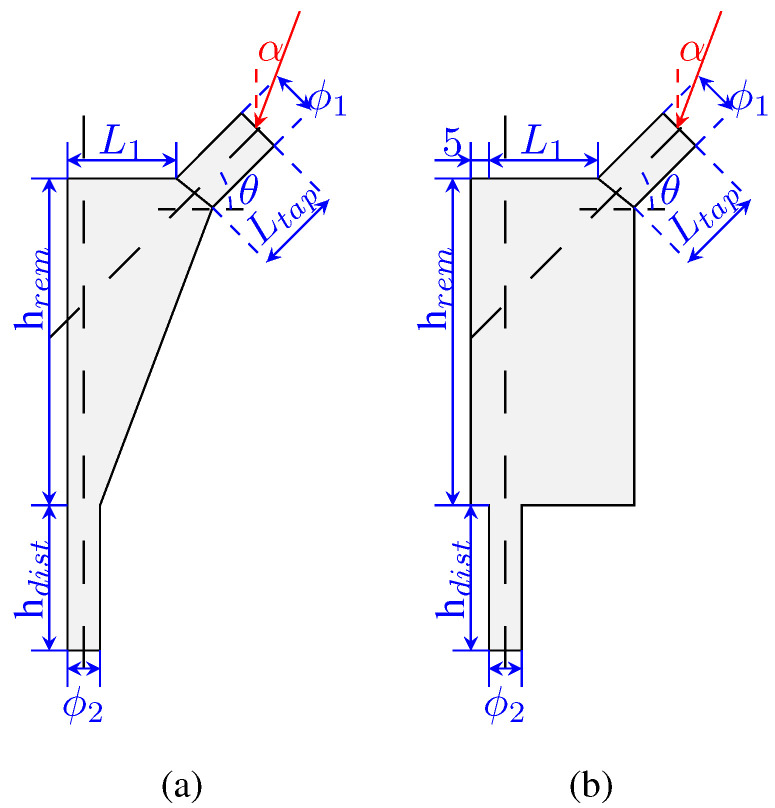
Design variables of the implant models: (**a**) for the conventional model meshed, and (**b**) for the extended model. All dimensions are in mm.

The design variables used to define the implant geometry are the following: $L_{1}$, $L_{tap}$, $\phi_{1}$, $\phi_{2}$, θ, $h_{dist}$, $h_{rem}$, and $h_{tap}$. The implant is composed of two solid parts (in the taper and in the distal-most part) and the remodeling area in the middle (where the implant will be porous). These areas are highlighted in Figure 1d. The process for the design variables selection followed a “sketching” rationale so that the model would be fully defined by these parameters. The design variables are shown in Figure 2a,b, indicating its meaning. Each design variable in the implant, as well as the angle α, has a range of values, which are presented in Table 1.

Then, with regards to these variables, it is possible to define the offset of the implant as

(5) $$offset=\left(l_{1}-\frac{\phi_{2}}{2}\right)+l_{tap}cos\left(\theta \right)+\frac{\phi_{1}}{2}sin\left(\theta \right).$$

The boundary conditions include the applied load (Figure 2) and a pinned constraint at the implant’s base. Each implant was modeled using Abaqus scripting and meshed with quadrilateral elements (Figure 1b,c). The resulting node and element data were then transferred to the in-house FEM code, which incorporates the remodeling algorithm. Additionally, an isotropic material model was assumed for the entire implant.

### 2.3. Neural Networks

This work used a type of neural network called a multi-layer perceptron in order to predict the optimal density distribution of the gyroid foam implant. The multi-layer perceptron consists of an input layer, one or more hidden layers, and an output layer. Each neuron receives inputs from the previous layer and applies a non-linear activation function to the weighted sum of the inputs.

(6) $$y=f\left(\sum_{i=1}^{n}w_{i}x_{i}+b\right),$$

where *y* is the output of the neuron, *f* is the activation function, $w_{i}$ represents the weights, $x_{i}$ represents the inputs, and *b* is the bias. The activation function is a non-linear function that introduces non-linearity into the network.

The input layer has a number of neurons equal to the number of input variables. Since the network does a pointwise prediction of the optimal density value, the input variables are the design variables of the model and the Cartesian coordinates $x_{i}$ and $y_{i}$ of the point being evaluated.

The number of hidden layers and the number of neurons in each hidden layer were determined systematically using a Bayesian optimization approach. The number of hidden layers was varied from 1 to 5 and the number of neurons were varied from 32 to 256. Additionally, three activation functions were tested, namely the hyperbolic tangent (*tanh*)

(7) $$tanh\left(x\right)=\frac{e^{2x}-1}{e^{2x}+1}$$

the rectified linear unit (*ReLu*)

(8) ReLu(x)=max(0,x)

and the sigmoid (*sig*) function.

(9) $$sig\left(x\right)=\frac{1}{1+e^{-x}}$$

The sigmoid function outputs values in the range (0, 1), making it useful for probabilistic interpretations, particularly in binary classification problems. However, it suffers from the vanishing gradient problem, where gradients become very small during backpropagation, slowing down or even halting learning in deeper networks.

Unlike the sigmoid function, *tanh* outputs values in the range (−1, 1), allowing it to represent both positive and negative activations. This makes it advantageous over the sigmoid function, as it centers the data around zero. However, it still experiences the vanishing gradient problem.

*ReLU* addresses the vanishing gradient problem seen in sigmoid and *tanh* functions by having a gradient of 1 for positive inputs. This leads to faster convergence during training. However, *ReLU* does not allow for negative values, meaning neurons with negative inputs become inactive, which can lead to the dying *ReLU* problem where neurons stop learning if they output zero consistently.

The output layer has a single neuron with a linear activation function. The output of the network is the predicted density value.

The training of the networks was performed using the mean squared error (MSE) as the loss function with the Adam optimizer.

(10) $$MSE=\frac{1}{n}\sum_{i=1}^{n}{\left(y_{i}-{\hat{y}}_{i}\right)}^{2}$$

where *n* is the number of samples, $y_{i}$ is the predicted value, and ${\hat{y}}_{i}$ is the target value. Using the MSE as the cost function penalizes the lower errors less than the higher errors, leading to faster convergence.

The batch size was also tuned, varying from 32 to 256. Batch training strikes a balance between computational efficiency and stable gradient estimates. The tuning was performed aiming at the lowest validation mean absolute error (MAE):

(11) $$MAE=\frac{1}{n}\sum_{i=1}^{n}\left|y_{i}-{\hat{y}}_{i}\right|$$

The networks were created with *keras* and the tuning was performed using *keras-tuner* (https://keras.io/, accessed on 6 April 2025). During the tuning, the networks were trained for 10 epochs. The model with the lowest validation MAE was selected for further training for 100 epochs; however, in order to avoid overfitting, an early stop criterion was defined where the training would be stopped when the validation loss did not decrease for 5 consecutive epochs.

### 2.4. Training Datasets

The training datasets were composed of 200 instances of the implant design, having created a dataset for the conventional model and a dataset for the extended model. For each instance, the information of all integration points is added to the dataset so that the total number of points of the dataset is as follows:

(12) $$total\;points=\sum_{i=1}^{n_{instances}}n_{points\;instance}$$

The dataset was split into 85% for training and 15% for validation. The testing dataset was composed of an additional 200 instances evaluated after the training.

**Figure 3:**
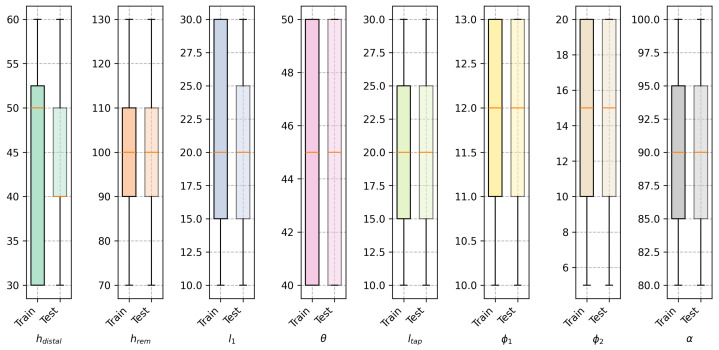
Distribution of the design variables in the training and testing datasets.

The instances in the training and testing datasets were generated by varying the design variables within the range of values shown in Table 1. For both sets it was certified that the combination of variables was unique. Figure 3 shows the distribution of the design variables in the training dataset for both models, where it is shown that for both datasets the design variables are uniformly distributed.

To facilitate training, each variable was scaled to the range [0, 1]:

(13) $$x_{scaled}=\frac{x-x_{min}}{x_{max}-x_{min}}$$

Scaling input features is crucial because it helps to maintain numerical stability when passing data through activation functions. Large, unscaled values can cause these functions to saturate, making gradients vanish and slowing or halting learning. Normalizing inputs leads to faster convergence and more reliable training since it avoids extreme outputs from activation functions. To avoid data leakage, the scaling was performed using the training dataset only. During training, the validation data were also scaled using the same scaling factors as the training data. The output variable is naturally scaled since it is a volume fraction value and, by itself, presents values between 0 and 1.

## 3. Results

The best hyperparameters for the neural networks were determined using a Bayesian optimization approach.

For the network trained with the conventional model dataset, the best hyperparameters comprised five hidden layers with 128 neurons and *ReLu* activations. Regarding the network trained with the extended model dataset, the best hyperparameters comprised five hidden layers with 128 neurons and sigmoid activations. Then, the best networks found for both models were retrained for up to 100 epochs or until the early stop criterion was met. During this training, the model considered the iteration with the best weights as the final model.

**Figure 4:**
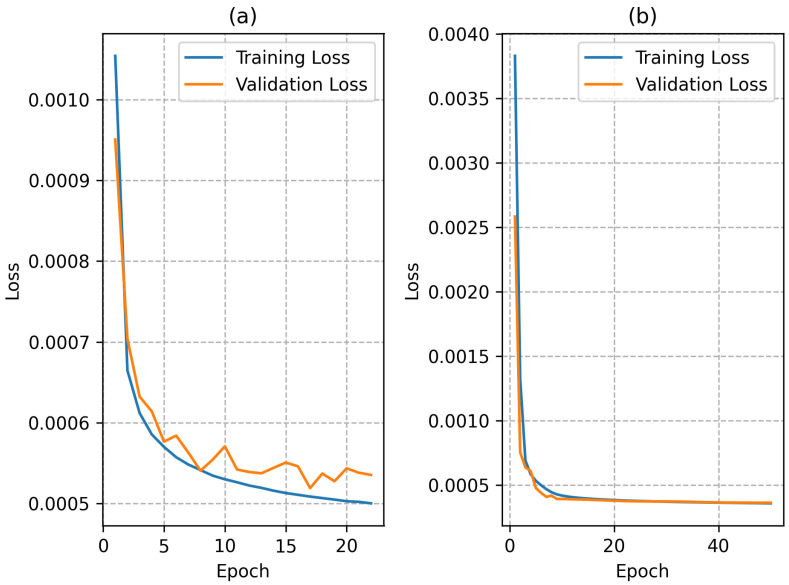
Training plots for both networks: (**a**) conventional model, (**b**) extended model.

The training plots for both networks during the retraining stage are shown in Figure 4. The use of a *ReLu* activation function in the hidden layers led to a faster convergence of the network trained with the conventional model dataset. The network trained with the extended model dataset had a slower convergence but the final loss was lower than the network trained with the conventional model dataset; this was most likely due to the larger training dataset, since each instance presents a larger number of points in the domain.

**Figure 5:**
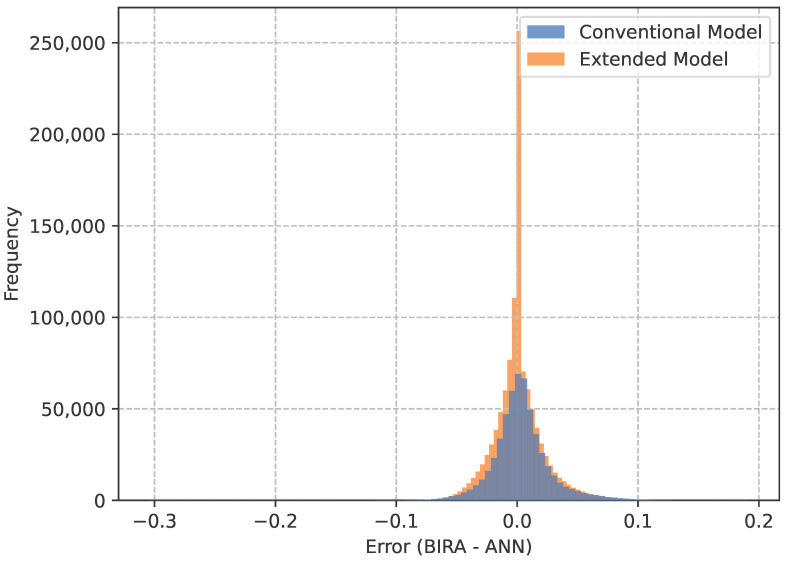
Histogram of the difference between the predicted and the target density for both networks.

In order to further analyze the performance of the neural networks, the absolute error between the predictions and the targeted value for each evaluation point in each instance of the testing dataset was plotted in the histogram shown in Figure 5. It is shown that for both networks, the median error in prediction was nearly zero. The majority of points are predicted with an error of less than 10% of the target volume fraction. The main difference between the neural network prediction in both cases is the smoother density field and some of the differences caught in the histogram are most likely due to this.

**Figure 6:**
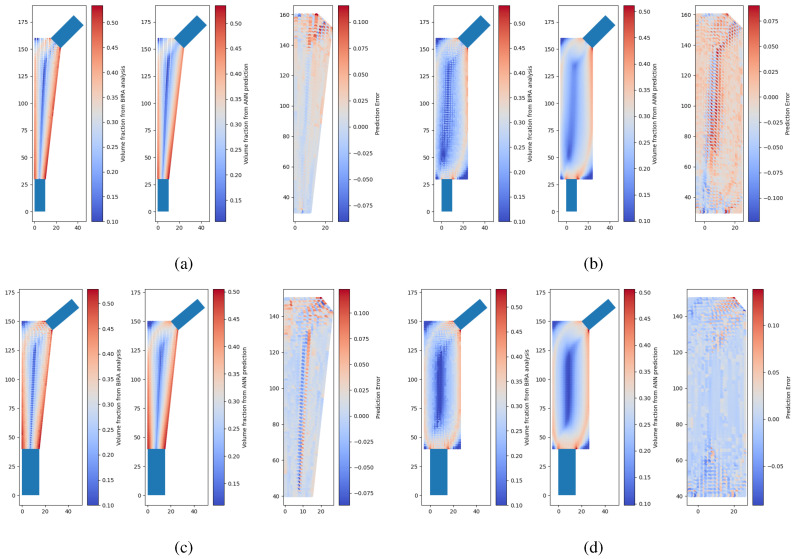
Two test set examples, where each example corresponds to an implant generated by a different combination of input features: (**a**) test case 1, conventional model; (**b**) test case 1, extended model; (**c**) test case 2, conventional model; (**d**) test case 2, extended model.

Some examples of neural network predictions for the conventional model and extended model are shown in Figure 6, where there are two instances of the testing set where each instance corresponds to a model with a unique combination of design variables. It is possible to observe that the neural network outputs a smoother density field than the model calculated through optimization with the remodeling algorithm and the FEM. The difference scatter between prediction and target shows that the difference is generally less than 0.1. It should be noted that these density distributions are then interpolated to design a porous implant. More details on this will be provided in the following sections.

Finally, there are significant differences between the optimized density in the conventional model and in the extended model. The main factor is if no anatomical domain constraint is used in the optimization analysis, then the algorithm by itself will not converge to the same gradient of density. Therefore, it is mandatory that the anatomical constraint is translated into the allowable design domain in the optimization approach. For this reason, in the following sections, only the performance of the conventional model is studied.

## 4. Strain Shielding Analysis

One main concern in the design of orthopedic implants is the stress shielding effect. Stress shielding is cause by the abrupt modification of the stress field on the bone tissue due to the inclusion of an implant, leading to modifications of the bone tissue density at the implant vicinity. Generally, such stress field modification is a consequence of the mismatch between the stiffness of the implant and the stiffness of the bone it is replacing. One of the most severe consequences of the strain shielding effect is that can lead to bone resorption and implant loosening.

In percentage terms, the stress or strain shielding effect is estimated through the quantification of the difference in strain in the bone before and after the insertion of the implant [34,35]

(14) $$\bar{\phi }=\frac{\phi_{\mathrm{intact}\;\mathrm{bone}}-\phi_{\mathrm{implanted}\;\mathrm{bone}}}{\phi_{\mathrm{intact}\;\mathrm{bone}}}$$

where $\bar{\phi }$ is a some measure of stress or strain in the bone. Some examples include the von Mises stress [21] or strain [36]. Due to the strain-driven nature of bone remodeling, a strain measure was used in this work. The equivalent strain measure was the von Mises strain. The change in density is determined by comparing the value of $\bar{\phi }$ to a reference stimulus *s* [34,35], which for the proximal femur was considered as *s* = 0.6 according to the literature [36]. Therefore, points where the values of $\bar{\phi }$ are lower than −s are considered as bone formation, and points with values of $\bar{\phi }$ higher than *s* are considered as bone resorption. Between −s and *s*, the bone is considered to be in the denominated “lazy zone” where bone formation and bone decay are in equilibrium.

Each model in the testing dataset was assessed for strain shielding by comparing an intact bone model with three implanted variants: a solid implant, a porous implant with uniform density, and a porous implant featuring a density distribution predicted by a neural network. The strain in each implanted model was evaluated against that of the intact proximal femur model to calculate a value of $\bar{\phi }$. The properties of all models are detailed in Table 2. The magnitude and direction of the applied load were selected to approximate similar studies [36] in accordance to ISO 7206-4 [37], involving an axial downward force in the y-direction applied to the taper of the implanted models and to the femoral head of the intact model. Additionally, the x-coordinate of the load application was kept consistent across both the intact and implanted models to ensure uniformity in testing conditions, guaranteeing a fair comparison of strain values. Due to the high variability in model geometry, the stem axis is aligned with the principal axis and no angle was considered in the application of the load.

**Figure 7:**
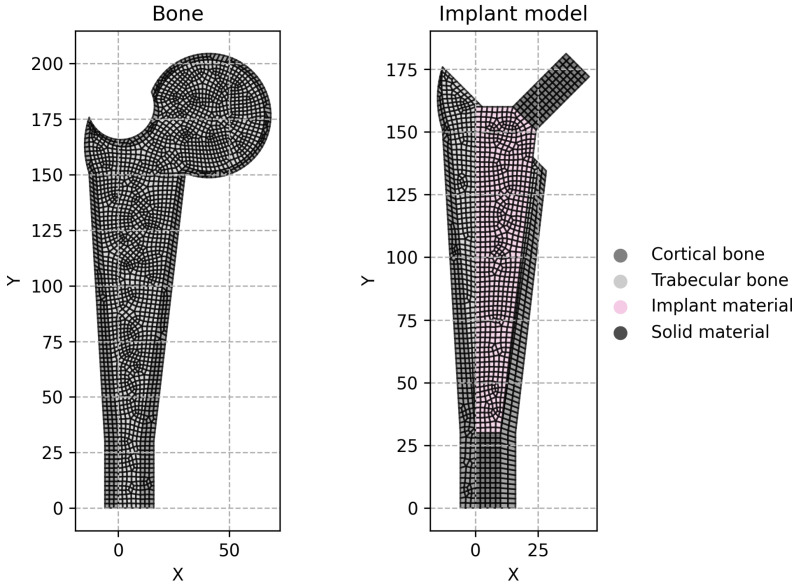
Computational models for the intact bone and implanted bone used for strain shielding analysis.

Figure 7 illustrates representative computational models used in this analysis, comparing an intact bone model with one containing an implant. The region designated as the implant material depends on the chosen implant design. If a solid implant is selected, the implant has zero porosity and the properties are the same for the entire model and equal to the solid material properties. For implants featuring graded porosity, the material properties are determined using the neural network-based approach. In the case of uniformly porous implants, each element is assigned the average density determined for the graded model with the same design features.

The performance of the implants was assessed based on their ability to reduce bone decay, stimulate bone growth, and withstand maximum load-bearing capacity. To evaluate load-bearing capacity, a 10 mm vertical displacement was applied at the same location of load application. Both the solid and porous sections of the implant were modeled as perfectly plastic, with a yield point defined as a function of its apparent density, according to (4). The properties of the porous section were derived through the homogenization of the gyroid foam structure, as detailed previously in the work. The stress shielding and strength analyses were performed using Abaqus 2023.

### Results and Discussion

**Figure 8:**
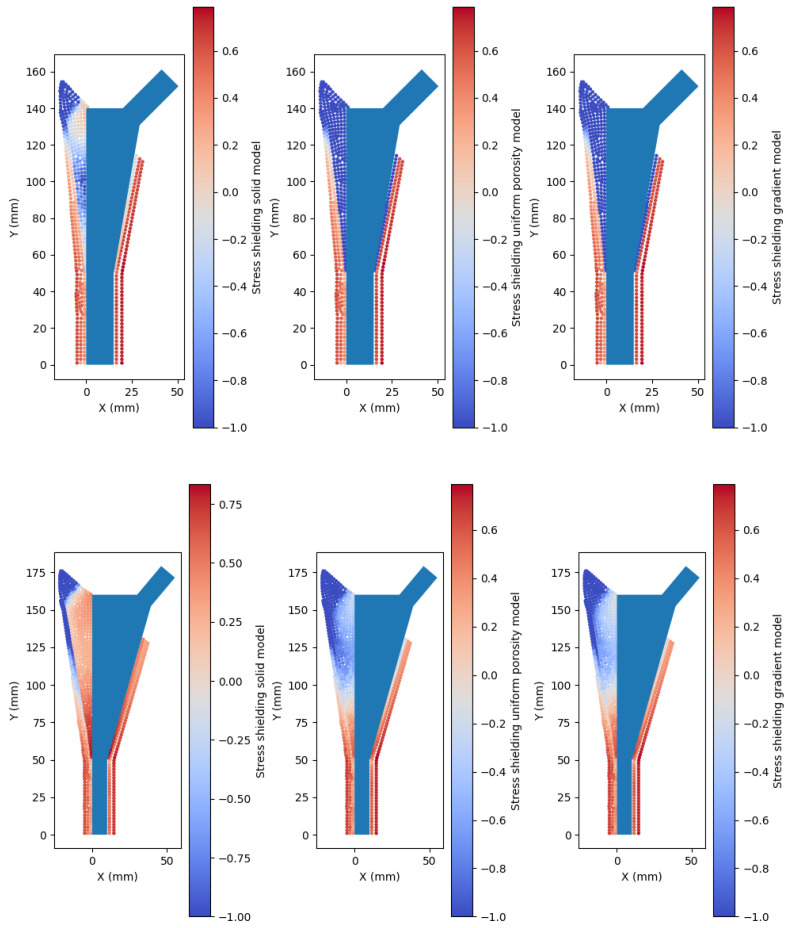
Strain shielding analysis on two instances of the testing set. The red in the color map indicates a tendency for bone resorption, while the blue indicates a tendency for bone formation.

From the analysis of the implanted models and comparison to the intact bone model, it was possible to access the tendency for bone resorption or bone growth in the implanted models in the porous or solid implants. Figure 8 shows an example of two instances in the testing set where the advantage of the porous implants is clear in comparison to a solid implant. Out of the total 200 instances in the testing set, only 170 led to geometrically feasible models. Due to the impossibility of showing the results for every model, an overall analysis of the entire set is presented.

**Figure 9:**
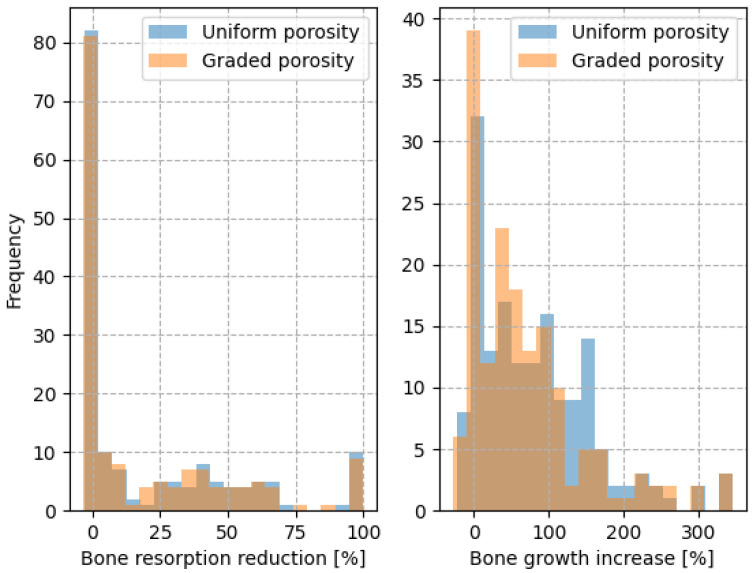
Bone resorption decrease and bone growth increase in the porous models compared to the solid model in the testing dataset.

A histogram comparing the bone resorption reduction and bone growth increase of the porous implants compared to the solid implants is shown in Figure 9. The porous models tend to reduce the amount of decaying bone and increase bone growth in comparison to a solid model. This evaluation is performed on the entire model domain and not on specific Gruen zones due to the fact that the implant and bone geometries present a high variability making it difficult to specify any trend.

From the analysis of the histogram, it can be concluded that among the uniformly porous models 50% exhibited reduced bone resorption and 81% demonstrated enhanced bone growth compared to the solid model. For the graded porous models, 50% showed reduced bone resorption and 77% exhibited improved bone growth compared to the solid model.

**Figure 10:**
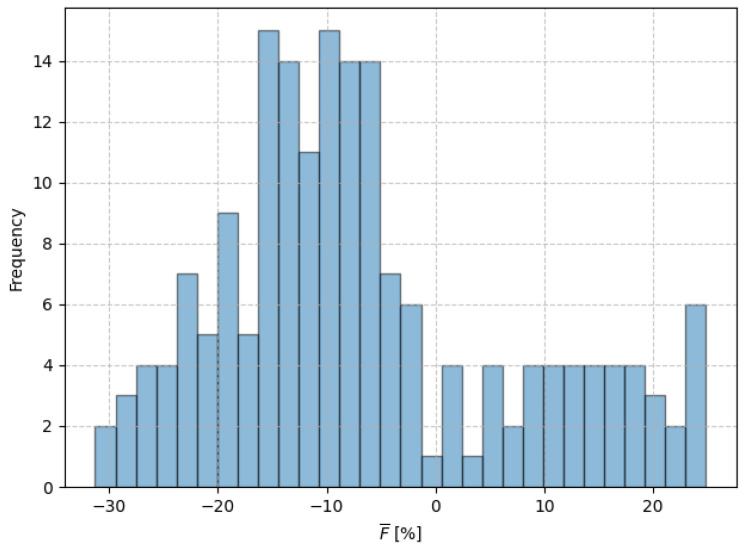
Load-bearing capability difference between the graded and uniformly porous implants in the testing dataset.

In general, uniformly porous implants and graded porous implants perform similarly in terms of preventing bone decay and stimulating bone growth, with no statistically significant differences observed in bone decay prevention (p=0.79) or bone growth stimulation (p=0.10). However, graded porous implants have the additional advantage of greater strength. The strength analysis shows that graded porous implants have a significantly higher load-bearing capability (p<0.05) compared to uniformly porous implants, as illustrated in Figure 10, which shows a histogram of the percentage difference in load-bearing capability between the graded and uniformly porous implants, calculated as follows:

(15) $$\bar{F}=\frac{F_{\mathrm{uniform}}-F_{\mathrm{graded}}}{F_{\mathrm{uniform}}}$$

where *F* denotes the load magnitude in [N] at the yield point when testing the implants under the same load case, as the strain shielding analysis defined in ISO 7206-4. From this analysis, it is possible to understand that 67.5% of the graded porous implants have a higher load-bearing capability than the uniformly porous implants, with improvements up to 20% while maintaining approximately the same mass.

There are several limitations to this approach in the evaluation of strains in the implanted model. The main limitation is the use of a plane strain approach. Even though generally it would not affect the results of the implant remodeling analysis and might even provide the advantage of obtaining a more intricate density field for the implanted model, this is not the case. The plane strain approach is a simplification of the three-dimensional problem where the strain in the z-direction is considered to be zero. This approach is valid when the thickness of the model is much larger than the other dimensions. In this case, the thickness of the model is 35 mm, which was applied to the whole model. Obviously, the implant is also surrounded by bone in the z direction, so the model lacks accuracy here. However, this approach was chosen to simplify the analysis and to allow for a faster computation of all the models in the testing dataset. Additionally, since the ISO 7206-4 standard was used to define the load case in strain shielding evaluation, the absence of the tendon in the greater trochanter leads to a insufficient and inaccurate loading in the lateral direction, which makes this area of bone less stressed and consequently prone to decay. This fact was verified in several of the tested models. Nevertheless, the strain analysis is comparative and the same simplification was applied to all models, so the effect in the estimation of a strain shielding value would be acceptable.

Another limitation of our work is the assumption of a perfectly plastic material model. Previous studies have shown that the equivalent plastic behavior of this cellular material exhibits hardening, particularly at higher volume fractions. However, at this stage, our focus is solely on the onset of yield to draw conclusions about the improvement in load-bearing capability for an implant with graded porosity. Moreover, the bone tissue was assumed to be isotropic and homogeneous. This assumption was made because a synthetic dataset was used, without medical imaging to infer trabecular arrangement, and to ensure a fair comparison across all models in the test set. However, variations in bone heterogeneity can result in different structural responses, possibly leading to different conclusions.

Material models were also simplified for the mechanical properties of Ti6Al4V in the homogenization model used in the simulation. If a prototype is manufactured, the properties of additively manufactured (AM) parts of this alloy may vary. Additionally, the crystallographic texture of 3D-printed titanium materials can significantly influence the results.

It is important to mention the fact that the optimization analysis considered just one type of initial domain. Different initial design domains, granted that anatomical constraints would be taken into account, will lead to different optimized configurations and consequently different strain shielding and strength results. For example, in the study by Bieger et al. [38], three femoral stem implants were compared for stability and stress shielding. In their study, it was concluded that the implant geometry selected for this work, with a longer stem, led to greater stress shielding than the other two designs with shorter stems. Additionally, another work by Liu et al. [39], using porous models of the same femoral stem implants as in the study by Bieger et al. [38], concluded that the longer stem was the least effective in stress shielding reduction compared to porous designs with the shorter stems. In some zones of the femur, the porous designs with the longer stem led to a higher stress shielding than the solid implant. This is a clear indication that the initial design domain has a significant impact on the optimized configuration and consequently on the strain shielding results.

One of the main simplifications performed in this work is the fact that the bone–implant interface was obtained via a conforming mesh of the two components. Also, the stress shielding effect was analyzed using the equivalent homogenized model of the gyroid foam instead of the accurate model, allowing for faster computation. Nevertheless, the present simplifications allowed us to come to the same conclusions as the study developed by Ziaie et al. [21], who realistically modeled the gyroid porous material and the bone–implant interface. In their work, the uniform gyroid foam was found to be more effective in stress shielding reduction with the disadvantage of not meeting mechanical strength requirements. Additionally, in their work, the gyroid foam was found to the preferable to other TPMS, namely diamond. Different conclusions were drawn in a different work by Wang et al. [18], where the graded structure was found to be more effective in stress shielding reduction than the uniform structure, even though a different unit cell topology and gradient strategy was used, suggesting that these strategies decrease implant stiffness more effectively than the remodeling approach.

While this study lacks experimental validation, the homogenized material law demonstrated strong correlation with various studies in the literature, spanning materials from polymers to metals and encompassing multiple additive manufacturing (AM) processes [33]. However, the comparison between solid, uniformly porous, and graded porous implants should still be supported by experimental validation. Specifically, the manufactured implant—only feasible through AM due to its porous interior—should be implanted into cadaveric bone and tested under conditions similar to those in studies such as [40,41].

In addition, further research is needed to explore the clinical implications of the optimized implants, as no biocompatibility study was conducted. This study focuses on a computational design approach aimed at enhancing the mechanical properties of the implant to reduce stress shielding while maintaining structural integrity. Nevertheless, the comparison between porous and solid implants in this study reinforces the claim that a porous design would outperform the current solid design of a commercial implant.

The main advantage of the approach would be the reduction in computational cost for inference after network training. On average, regarding the conventional model, the simplified 2D model took 120 s for the optimization stage, while training took 570 s and inference with the trained network takes on average 0.18 s. For the extended model, these values were on average 456 s, 2900, and 0.29 s. Also, in terms of design flexibility, this approach can be patient specific, as the input variables can be determined from the analysis of patient medical imaging and the ANN will suggest a porous implant with improved properties. Table 3 shows a comparison between implant design approaches in terms of applicability to patient specific design, efficacy, and computational cost.

## 5. Optimized Models

From the optimal density distribution, it is possible to derive a three dimensional model of the implant with the gradation of the density of the gyroid foam. This is achieved by extruding the density map to create a three-dimensional point cloud, which encodes spatial information about the volume fraction of the gyroid foam. The gyroid surface equation is given by

(16) $$G:\sin\left(\frac{\pi x}{L}\right)\cos\left(\frac{\pi y}{L}\right)+\sin\left(\frac{\pi y}{L}\right)\cos\left(\frac{\pi z}{L}\right)+\sin\left(\frac{\pi z}{L}\right)\cos\left(\frac{\pi x}{L}\right)=0$$

which is a surface that divides the three dimensional space into two equal volume regions, and where *L* denotes the size of the unit cell. The density variation is obtained by thickening the gyroid surface with a value of *t* in the direction of the normal vector to the surface. Therefore, the gyroid foam corresponds to the space enclosed by the two surfaces

(17) $$t_{1}\leq G\leq t_{2}$$

where $t_{1}=-t/2$ and $t_{2}=t/2$.

In a gyroid foam, the apparent density and the thickness are related linearly according to

(18) $$\rho_{app}=2.868\frac{t}{L}$$

The remodeling algorithm outputs only a homogenized density, while the unit cell size is defined during post-processing. Pore size and porosity are key parameters influencing biological phenomena such as bone ingrowth. These must be carefully chosen to ensure both biological effectiveness and manufacturing precision [8].

In order to create the three-dimensional models, the thickness value of each point in the surface is inherited from the integration point at the lowest Euclidean distance to that point. The surface is plotted in the three-dimensional space into a triangular mesh. Each point in the traingulation of the mesh is then assigned the density value of the integration point with the lowest Euclidean distance. Finally, the solid areas are added to the model, which can be 3D printed.

**Figure 11:**
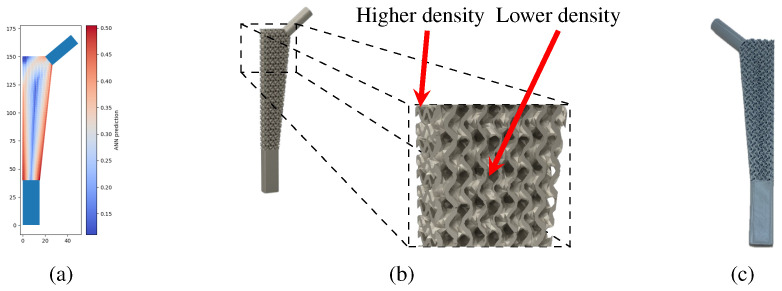
Optimized stem model based on the gyroid foam structure: (**a**) density distribution obtained from the neural network and (**b**) three-dimensional model with density gradation, showing the differences in density through thickness for a structure with a unit cell size of 5 mm. (**c**) Three-dimensionally printed prototype.

Examples of the density gradation of the gyroid structure in a three-dimensional model can be seen in Figure 11, which shows the reference density distribution solution and the renderized model with the density gradation in the remodeling area.

A prototype of the model shown in Figure 11b is shown in Figure 11c. This model was manufactured with PLA filament using a fused filament fabrication (FFF) printer, the Prusa MK3s.

Manufacturing implants presents a significant challenge. Due to mechanical strength requirements, only metals or ceramics can be used, and these materials are predominantly processed through powder bed fusion or binder jetting. In powder-based methods, removing residual powder from the porous structure presents a challenge [42]. Pore size must be carefully optimized to support bone growth and cell proliferation while remaining large enough to allow effective powder removal. An alternative approach is metal fused filament fabrication (FFF). In this process, the filament is loaded with metal particles, which is extruded to form the part. After extrusion, the binder is removed, and the part undergoes sintering. Some difficulties that emerge here are the removal of the binder and sintering [43], which become more complicated for thicker parts. Also, even though gyroid structures are nearly self-supporting—and are printed without supports due to pore size constraints—the resulting structures still exhibit considerable imperfections when the overhang angle is too low.

## 6. Conclusions

This work presented an approach which leverages the use of neural networks to predict the optimal density distribution of a gyroid foam implant. It highlighted the need for an anatomically constrainted design domain, since otherwise the model does not tend towards an optimized solution fitting the inside of the femur. It was concluded that a sufficient sampling of optimized density fields was successful in modeling the density for cases not explicitly seen during training. Then, the optimized designs were tested for strain shielding and load-bearing capability. For this purpose, uniformly porous and graded porosity implants were compared to a solid implant. The results showed that the porous implants tend to reduce bone decay and stimulate bone growth in comparison to the solid implant. Regarding strain shielding reduction, there was no significant difference between the uniform and graded porosity designs, having both shown improvements in comparison to the solid design. However, due to the nature of the remodeling procedure, the optimized design presents better strength than the uniform porosity design. Finally, a manufacturable prototype was suggested which replicates the variations in the volume fraction of the gyroid foam and several manufacturing aspects were analysed.

This work advances the patient-specific modeling of porous femoral stem implants by enabling input variables to be derived from patient imaging, resulting in implants that better align with individual anatomical structures. Future research directions can include the expansion of the dataset for different implant types (i.e., short stem) and different ML models.

## Figures and Tables

**Table 1:** Range of values for the design variables.

	$L_{1}$	$L_{\mathrm{tap}}$	$\phi_{1}$	$\phi_{2}$	θ	$h_{\mathrm{dist}}$	$h_{\mathrm{rem}}$	$h_{\mathrm{tap}}$	α
Range	10–30	10–30	10–13	5–20	40–50	30–60	70–100	10–30	80–100
Levels	5	5	4	4	3	4	4	5	5

**Table 2:** Properties of the models used in strain shielding analysis.

$E_{\mathrm{Ti}-6\mathrm{AL}-4V}$ [GPa]	$E_{\mathrm{cortical}}^{\mathrm{bone}}$ [GPa]	$E_{\mathrm{trabecular}}^{\mathrm{bone}}$ [GPa]	Cortical Thickness [mm]	$\nu_{\mathrm{bone}}=\nu_{\mathrm{implant}}$	Plane Strain Thickness [mm]	Load Magnitude [N]
110	17.5	5	6	0.3	35	2300

**Table 3:** Comparison between implant design approaches.

Approach	Computational Cost	Efficacy	Patient Specific	Works
ML-based surrogate model (i.e., ANN, SVM)	Accelerates optimization by surrogating the calculation of objective function (i.e micromotion, stress shielding) to ML.	Selected implant design features that fit the objective (minimization of micromotion or stress shielding).	Surrogate model should be calibrated for each patient anatomy or use a statistical femur model.	[24,25]
Topology optimization	Iterative runs of FEM or other analysis.	Achieves a structure with reduced mass yet structurally safe. Improves stress shielding indirectly.	Runs optimization on each implant.	[21]
ANN	Surrogates topology optimization.	Improves stress shielding indirectly.	Input features for implants are selected for each patient.	This work.

## Data Availability

No new data were created or analyzed in this study. Data sharing is not applicable to this article.
